# Exploring the Potential of Multispectral Imaging for Automatic Clustering of Archeological Wall Painting Fragments

**DOI:** 10.3390/s26072111

**Published:** 2026-03-28

**Authors:** Piercarlo Dondi, Lucia Cascone, Chiara Delledonne, Michela Albano, Elena Mariani, Marina Volonté, Marco Malagodi, Giacomo Fiocco

**Affiliations:** 1 Department of Electrical, Computer and Biomedical Engineering, University of Pavia, Via Ferrata 5, 27100 Pavia, Italy; piercarlo.dondi@unipv.it; 2 Centro Interdipartimentale di Studi e Ricerche per la Conservazione del Patrimonio Culturale (CISRiC), University of Pavia, 26100 Pavia, Italy; marco.malagodi@unipv.it; 3 Department of Computer Science, University of Salerno, Via Giovanni Paolo II, 132, 84084 Salerno, Italy; lcascone@unisa.it; 4 Department of Physics, University of Pavia, Via Bassi 6, 27100 Pavia, Italy; chiara.delledonne@unipv.it; 5 Arvedi Laboratory of Non-Invasive Diagnostics, Department of Musicology and Cultural Heritage, University of Pavia, Via Bell’Aspa 3, 26100 Cremona, Italy; michela.albano@unipv.it; 6 Independent Researcher, Via Cavour 6, 20851 Lissone, Italy; elenamariani99@gmail.com; 7 Sistema Museale “Cremona Musei” e Museo Archeologico, Comune di Cremona, Via Ugolani Dati 4, 26100 Cremona, Italy; marina.volonte@comune.cremona.it

**Keywords:** multispectral imaging, UV fluorescence, infrared reflectance, clustering, machine learning, deep learning, Roman wall painting

## Abstract

The digital reconstruction of damaged archeological wall paintings is a challenging task due to severe material degradation, high fragmentation, and the lack of reference images. A crucial preliminary step is the separation and grouping of fragments originating from different wall paintings, which are often found mixed together at archeological sites. To address this issue, we explored the potential of multispectral imaging (MSI) for unsupervised fragment clustering, aiming to assess whether integrating multiple spectral bands can enhance fragment discrimination compared to using the visible band alone. As a test set, we examined five groups of wall painting fragments from a Roman domus (1st c. BC–1st c. AD) provided by the Archaeological Museum of Cremona (Italy). Images were acquired using the Hypercolorimetric Multispectral Imaging (HMI) system developed by Profilocolore^®^ Srl (Rome, Italy). Specifically, we considered visible reflectance (VIS), infrared reflectance (IR), infrared false color (IRFC), and Ultraviolet-induced Fluorescence (UVF) images. Through a systematic benchmarking study, we compared several state-of-the-art feature extraction and clustering methods across single- and multi-band configurations. Results show that combining MSI data can substantially enhance the system’s ability to correctly separate and group fragments, indicating a promising direction for future research.

## 1. Introduction

Restoring and studying damaged wall paintings is a complex task that demands significant effort from restorers, archeologists, and art experts. Common difficulties include small and irregularly shaped fragments, chromatic alterations of pigments, and the loss of substantial portions of the pictorial surface [1]. Although these challenges affect any damaged wall painting, they are especially pronounced in archeological contexts. Moreover, whereas recently damaged works—such as medieval or Renaissance wall paintings affected by earthquakes or conflicts—may still rely on historical photographs to guide reconstruction [2,3], no such reference imagery exists for ancient Greek or Roman wall paintings [4,5,6].

Over the years, scientists have proposed various solutions to assist restorers and archeologists. While physical–chemical analyses remain standard tools for wall painting investigations [7,8], a variety of computer vision methods also exist.

For example, some early approaches adopted contour-matching and curve-fitting techniques to solve 2D and 3D puzzles [9] and to reconstruct pottery [10], wall paintings [4], and archeological artifacts [11]. Other studies instead investigated how fragments naturally form [12,13,14] to simulate the plausible fragmentation of wall paintings.

Several image-based reconstruction methods rely primarily on chromatic cues [15], combine color and geometric descriptors [16,17], or employ deep learning techniques [18,19]. However, these approaches typically assume puzzle-like conditions, where fragments are all available and their boundaries align well—conditions rarely met in the restoration of wall paintings. More specialized solutions have therefore been developed for mural reassembly, combining both geometric and chromatic features [14,20]. Complementary advances have also emerged from 3D matching [21], photogrammetry [3,22], multi-feature matching [23,24], and genetic algorithms [25]. Additional contributions include SIFT-based pattern recognition [26], handcrafted feature descriptors [27], and, more recently, GAN-based approaches enabling self-supervised alignment even in the presence of gaps [28]. Nevertheless, a general solution capable of addressing all the inherent complexities of the problem is still lacking.

In this paper, we focus on a preliminary yet crucial phase of the restoration workflow: the correct subdivision of mixed fragments, a frequent issue at archeological sites. Previous studies treated this problem as a stylistic classification task, applying machine and deep learning methods to separate the fragments based on artistic style, either using synthetic [29,30] or real datasets [31]. A different approach, tested on Pompeii’s wall paintings, instead involved the use of semantic motif segmentation [6].

The main novelty of this work lies in the use of multispectral imaging (MSI) for unsupervised fragment clustering. MSI is widely applied in the cultural heritage field, for example, to investigate painted artworks and obtain information on materials, execution techniques, and conservation conditions through a non-invasive approach [32,33,34,35]. While various works have employed MSI techniques for the study and characterization of wall paintings [36,37], to the best of the authors’ knowledge, MSI has not yet been applied to the clustering of archeological wall painting fragments. This paper aims to explore the potential of MSI for this task, assessing whether integrating multiple spectral bands can enhance fragment discrimination compared to visible imaging alone.

As for the test data, various real-world collections of wall painting fragments exist, even if only a few are publicly available. Notable examples include photographic datasets from the Ovetari Chapel [2], Akrotiri [4], and Petra [11] and 2D/3D acquisitions from Thera, Tongeren, and Kerkrade [21,23,38]. Other contributions are the digitization of the Severan Marble Plan [5], photogrammetric surveys in Cyprus [39], and the recent RePAIR dataset containing 2D/3D data of Pompeian wall painting fragments [40]. Synthetic datasets have also been proposed in the scientific literature, including digitally fractured murals from Dunhuang [41], the large-scale DAFNE dataset [1], the CLEOPATRA collection [30], and the POMPAAF dataset [31]. However, to the best of the authors’ knowledge, to date, there is no publicly accessible dataset of multispectral images of archeological wall painting fragments.

To conduct the experimentation, we therefore acquired MSI data of a set of wall painting fragments originating from a Roman domus (1st century BC–1st century AD), generously provided by the Archaeological Museum of Cremona (Italy). Specifically, we considered four multispectral image types: visible light reflection (VIS), infrared reflection (IR), infrared false color (IRFC) and Ultraviolet-induced visible Fluorescence (UVF). All these techniques are commonly employed for the examination of artworks to document surface conditions, reveal subsurface features [33,35,42], characterize pigments [32,33], map varnishes and detect conservation treatments [43].

After the image acquisition, we compared several state-of-the-art feature extraction methods and clustering algorithms across both single- and multi-band configurations to evaluate our hypothesis regarding the advantages of MSI over visible imaging alone.

The remainder of this paper outlines the acquisition process, the dataset, and the clustering strategy employed. It then describes the experimental setup and summarizes the main results. The work concludes with a critical discussion of the findings, emphasizing key insights and limitations, and with the suggestion of some possible future developments.

## 2. Materials and Methods

### 2.1. Image Acquisition

The image acquisition was performed using the Hypercolorimetric Multispectral (HMI) system [44,45] developed by Profilocolore^®^ Srl (Rome, Italy). The system is based on a modified Nikon Z8 mirrorless digital camera (Minato, Tokyo, Japan) equipped with its built-in 45.7-megapixel sensor. The modification extends the sensor spectral sensitivity of the silicon sensor over the approximate range of 300–1000 nm. For the acquisition, the camera was equipped with an FTZ adapter an FTZ mount adapter (Nikon Corporation, Tokyo, Japan) and an AF-S Nikkor 50 mm f/1.4 G lens (Nikon Corporation, Tokyo, Japan, manufactured in China). Illumination was provided by two Godox TT600 xenon flashes (GODOX Photo Equipment Co., Ltd., Shenzhen, China) featuring broadband emission across the 300–1000 nm range and a color temperature of 5600 K. The flashes were used in combination with optical filters: filter A (400–700 nm) for the visible range and filter B (300–500 nm and 700–1000 nm) for the Ultraviolet (UV) and Near-Infrared (NIR) ranges. For color calibration, a custom 36-patch color checker supplied by Profilocolore^®^ was included in each acquisition. Image processing was performed using SpectraPick^®^ software (Profilocolore^®^), which exploited the two raw images acquired using filter A and filter B as input data to generate the VIS, IR and IRFC images.

UVF images were instead acquired using two Madatec CR230B-HP UV LED lamps (Pessano con Bornago, Milano, Italy), characterized by 365 nm wavelength and 3 W power, used in combination with filter A and a UV–IR cut filter (300–700 nm).

**Figure 1:**
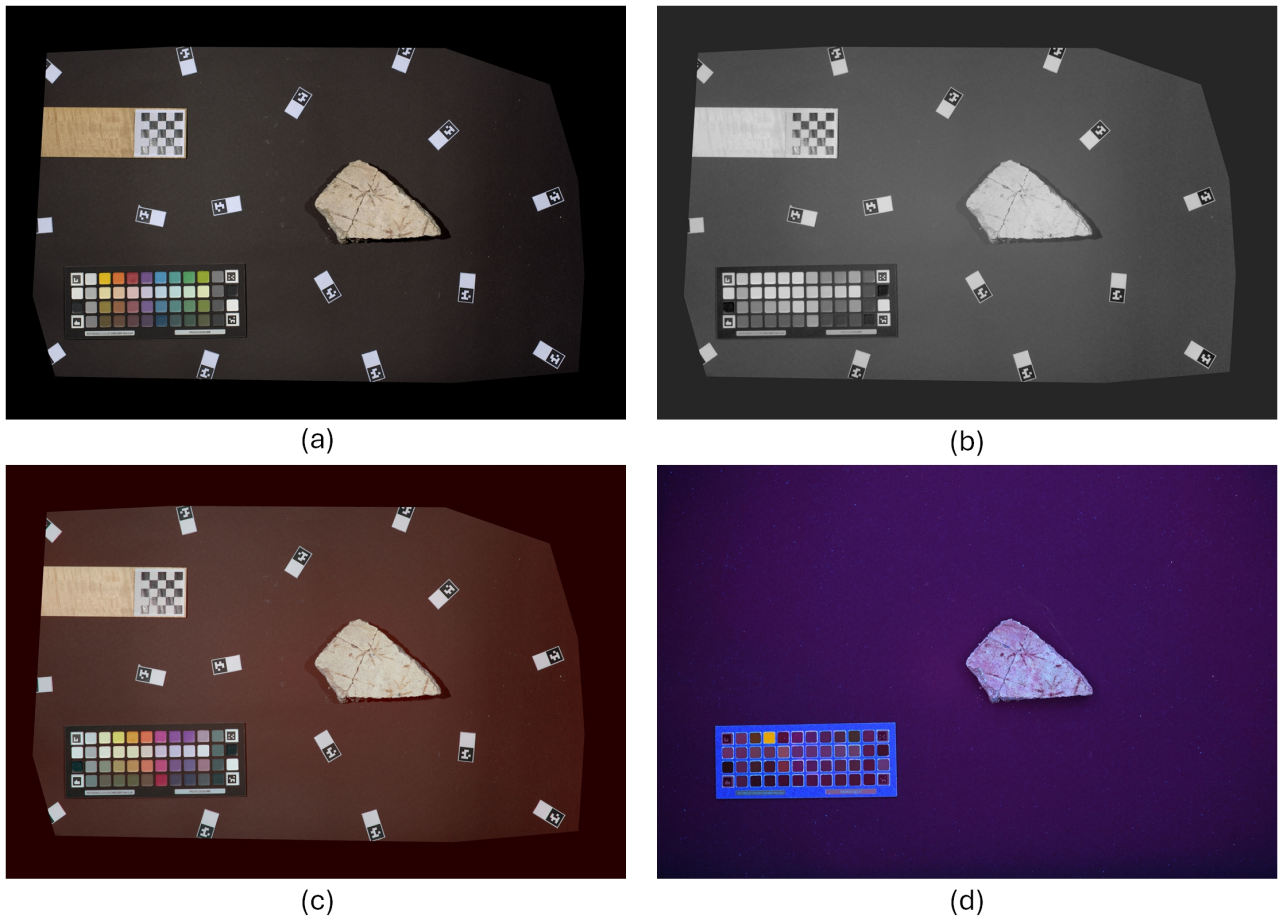
Example of the acquired multispectral image types for one sample fragment: (**a**) VIS; (**b**) IR; (**c**) IRFC; (**d**) UVF. Reference objects on the VIS image: top left, the metric marker; bottom left, the color checker; around the fragment, small markers used for flat field correction.

Figure 1 shows an example of the four multispectral image types for a sample fragment. The acquisition procedure was designed to guarantee that, for each fragment, the four images were perfectly aligned.

**Figure 2:**
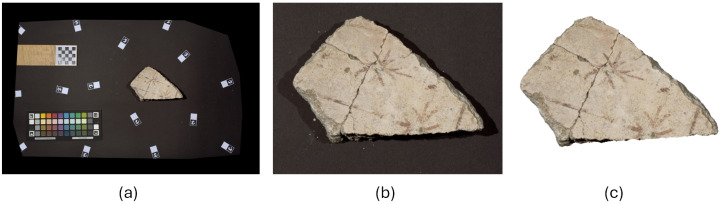
Example of image rescaling and cleaning: (**a**) original image with color checker and reference markers; (**b**) fragment rescaled and cropped; (**c**) rescaled fragment with background removed.

During postprocessing, each image was rescaled using the reference metric marker present in the photo. This operation ensured that all photographed fragments had the correct proportion to each other. The images were then cropped around the fragment and the background removed. Figure 2 shows an example of the cleaning process for a sample VIS image. The process was repeated in the same way for each image type.

### 2.2. Dataset

The dataset is composed of multispectral images of wall painting fragments provided by the Archaeological Museum of Cremona (Italy). The fragments come from a rich Roman domus (1st c. BC–1st c. AD) uncovered during archeological excavations conducted between 2005 and 2008 in Piazza Marconi in Cremona. Specifically, they decorated the walls of one of the domus bedrooms (*cubiculum*), nicknamed “Ariadne’s Room” due to the presence of paintings depicting the myth of the Cretan heroine. The collapse of the first-floor room during the sacking of Cremona in 69 AD resulted in the wall paintings being reduced to thousands of disconnected fragments, which were collected during the excavation in 1000 crates. Given the high number of fragments, they are still under examination by experts, and only a part of them has been restored and exhibited in the museum.

**Figure 3:**
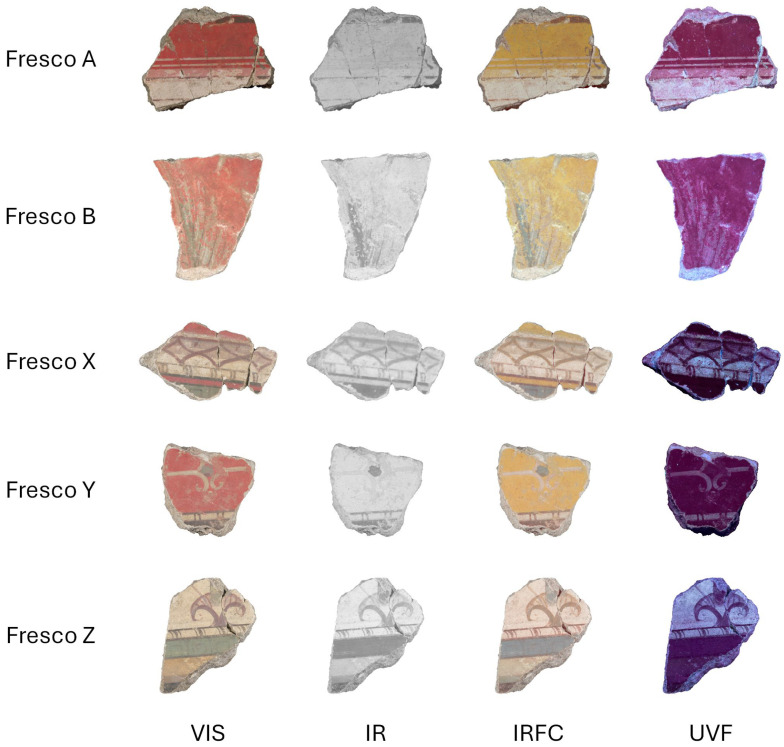
The four multispectral image types for a sample fragment of each wall painting set.

For the purpose of this exploratory study, we considered fragments that have been examined and classified but not yet recomposed. Among them, we selected, with the help of the museum curator and a Roman painting expert, a small group of 29 fragments representative of the problem. Specifically, we considered five sets of pictorial fragments, originating from different areas of the Ariadne Room’s walls, showing various colors and/or geometrical patterns. We instead excluded fragments that only had a uniform coloration without any pattern (e.g., all white) and thus could not be reliably assigned to a specific cluster. The sets are composed as follows: Set A contains 7 fragments; Set B contains 6; Set X contains 8; Set Y contains 5; and Set Z contains 3. Figure 3 shows the four multispectral image types for some sample fragments.

### 2.3. Problem Setup

Let S={VIS,IR,IRFC,UVF} denote the set of available multispectral image types. To simplify the notation, we define them as *bands* below.

Each fresco fragment i∈{1,…,N} is observed over the four bands as a collection of images ${\left\{I\left(i,s\right)\right\}}_{s\in S}$, where I(i,s)∈I denotes the image of fragment *i* in the band *s*. The goal is to partition a set of mixed fragments into *K* clusters based on their visual similarity across bands.

We denote by $y_{i}\in \left\{1,\ldots ,K\right\}$ the ground-truth fresco identity of fragment *i*, used only for evaluation. All images are associated with a binary foreground mask M(i,s), obtained during preprocessing, which isolates the painted fragment from the background. Unless otherwise specified, all feature descriptors are computed exclusively over masked pixels, ensuring that background regions do not contribute to the extracted representations. When required, images are converted to grayscale prior to feature extraction.

**Figure 4:**
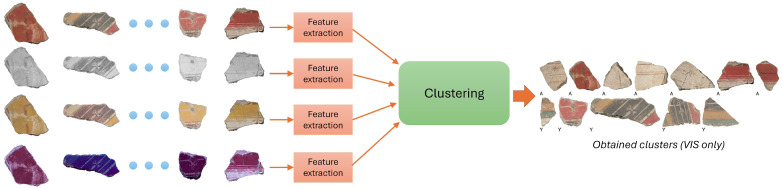
Overview of the clustering procedure.

Figure 4 summarizes the overall process. Note that even if all bands may contribute to the correct clustering, the output is generally reported only in visible light for better clarity, so that the restorers can visually compare the proposed clusters with the real fragments.

### 2.4. Multispectral Feature Representation

Let D denote a set of descriptor families. Each descriptor d∈D defines a feature extractor $\phi_{d}:I\to R^{m_{d}}$ that maps an input image to an $m_{d}$-dimensional vector. For each fragment *i*, a descriptor *d* is computed over a subset of bands $S_{d}\subseteq S$ and fused across bands by concatenation, as formalized in Equation (1). We use ‖ to denote vector concatenation.

(1) $$\Phi_{d}\left(i\right)\;=\;\underset{s\in S_{d}}{∥}\phi_{d}\;\left(I(i,s)\right)\;\in \;R^{|S_{d}|\;m_{d}}$$

When multiple descriptors are considered, their multispectral representations are combined into a single embedding $x_{i}$ by concatenation across descriptors (Equation (2)).

(2) $$x_{i}\;=\;\underset{d\in D}{∥}\Phi_{d}\left(i\right)\in R^{D}$$

The final embedding dimension is $D\;=\;\sum_{d\in D}\left|S_{d}\right|\;m_{d},$ where $m_{d}$ denotes the per-band dimensionality of descriptor *d* and $S_{d}\subseteq S$ is the set of bands used by *d*. The complete set of feature vectors is standardized across the dataset, enforcing zero mean and unit variance per feature dimension.

In this study, we consider one deep learning descriptor, based on a ResNet-18  [46], pretrained on ImageNet [47], and three handcrafted descriptors, namely Color Histograms, Local Binary Pattern (LBP) [48] and Gabor filter. As color spaces, we consider RGB, HSV and LAB for the bands VIS, IRFC and UVF, and grayscale for IR, since it is single-channel. Depending on the configuration, descriptors are used either individually or concatenated. The formal definition of each descriptor and the chosen hyperparameters are reported in Appendix A.

### 2.5. Clustering Approaches

Given the standardized multispectral feature embeddings, we evaluated several classical unsupervised clustering methods to group fresco fragments based on visual similarity. For fixed-*K* algorithms, the number of clusters *K* was set to match the number of distinct fresco groups in each dataset configuration. Hyperparameters were selected empirically through preliminary experiments and kept fixed across all runs.

We considered five clustering algorithms representative of different optimization paradigms:

- K-means [49] partitions the data by minimizing the within-cluster sum of squared distances to cluster centroids. We adopted the k-means++ initialization strategy [50] and performed multiple random restarts to reduce sensitivity to initialization.
- Spectral Clustering (Spec. Clust.) [51] constructs a *k*-nearest-neighbor graph in feature space and performs clustering in the spectral embedding derived from the normalized graph Laplacian, enabling the identification of non-convex cluster structures.
- Agglomerative Clustering with Ward linkage (Ward) [52] iteratively merges clusters so as to minimize the increase in total within-cluster variance, producing a hierarchical dendrogram that is cut to obtain exactly *K* clusters.
- Balanced Iterative Reducing and Clustering using Hierarchies (BIRCH) [53] performs incremental hierarchical clustering using a compact clustering feature tree, preserving local structure while maintaining computational efficiency.
- Density-Based Spatial Clustering of Applications with Noise (DBSCAN) [54] groups samples based on local density defined by a neighborhood radius ε and a minimum number of points. Unlike the previous methods, DBSCAN automatically determines the number of clusters and identifies isolated samples as noise. We report preliminary DBSCAN results but focus primarily on fixed-*K* methods to enable controlled comparisons across configurations.

All clustering methods operate directly on the *D*-dimensional feature vectors, without additional dimensionality reduction.

### 2.6. Cluster-to-Label Alignment

Clustering algorithms produce arbitrary cluster identifiers that are not directly comparable to ground-truth fresco labels. For visualization and error analysis, we align predicted clusters to ground-truth labels via optimal assignment.

Let $z_{i}\in \left\{1,\ldots ,K\right\}$ denote the cluster ID predicted for fragment *i* and let $y_{i}\in \left\{1,\ldots ,K\right\}$ be its ground-truth fresco label. Alignment relies on the contingency matrix $C\in N^{K\times K}$, whose entries measure the overlap between predicted clusters and ground-truth labels. In particular, Equation (3) counts the number of fragments assigned to cluster *k* and belonging to label *ℓ*.

(3) $$C_{k\ell }\;=\;\left|\left\{\;i\;:\;z_{i}=k\;\wedge \;y_{i}=\ell \right\}\right|$$

A one-to-one assignment π:{1,…,K}→{1,…,K} is then selected to maximize the number of matched fragments. The optimal mapping $\pi^{*}$ is obtained by solving the maximization problem in Equation (4).

(4) $$\pi^{*}\;=\;\arg\max_{\pi }\sum_{k=1}^{K}C_{k,\pi (k)}$$

The above objective can be solved efficiently through Hungarian matching, formulated as a linear sum assignment on an equivalent cost matrix. Aligned predictions are then computed as ${\hat{y}}_{i}=\pi^{*}\left(z_{i}\right)$, which enables per-fragment error flags and cluster visualizations using fresco label names.

This alignment step is introduced only for interpretability, namely visualization and error reporting. The quantitative clustering metrics reported in Section 3.2 are label-invariant and therefore do not require any label alignment.

## 3. Experiments

### 3.1. Experimental Procedure

The experiments were intended to evaluate the contribution of each multispectral image type (bands in the following) to the clustering. First, we considered each band separately, to obtain a baseline, then we tested all the possible combinations (two, three or four bands together). For each band or combination of bands we tested the deep learning-based feature extractor (ResNet-18) and all the combinations of handcrafted feature descriptors (histograms, LBP, Gabor).

**Figure 5:**
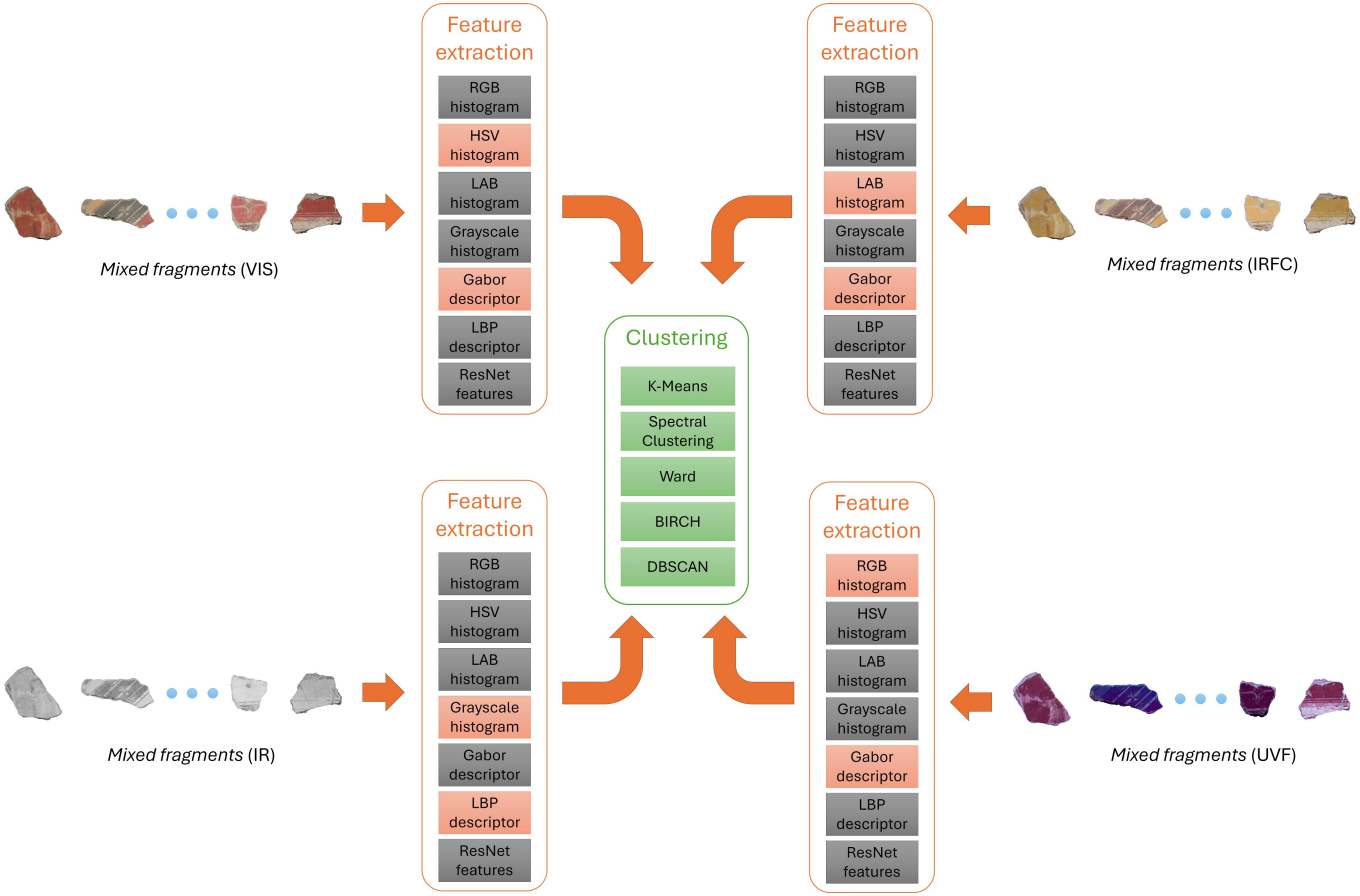
Example of experiment: One of the possible combinations of multispectral image types and feature extraction methods. Colored blocks are enabled; gray blocks are disabled.

For all configurations, we tested all four clustering methods considered. Figure 5 shows an example of a possible combination of bands and features tested.

In real-world scenarios, it is uncommon to find fragments from many different wall paintings mixed together. We therefore considered two common cases: (i) binary clustering, in which fragments from two sets are mixed, and (ii) ternary clustering, in which fragments from three sets are mixed. Since our dataset includes five fragment sets, there are ten possible combinations for both the binary and ternary settings.

### 3.2. Evaluation Metrics

To quantitatively assess clustering quality, we employ standard validation metrics that compare the predicted partition $Z=\{z_{1},\ldots ,z_{N}\}$ with the ground-truth partition $Y=\{y_{1},\ldots ,y_{N}\}$ while correcting for chance agreement.

Using the contingency matrix $C\in N^{K\times K}$ defined in Section 2.6, where $C_{k\ell }$ counts fragments in cluster *k* with ground-truth label *ℓ*, we define the marginals $a_{k}=\sum_{\ell =1}^{K}C_{k\ell }$ and $b_{\ell }=\sum_{k=1}^{K}C_{k\ell }$. We focus on two widely adopted metrics: the Adjusted Rand Index (ARI) and the Adjusted Mutual Information (AMI).

ARI measures partition similarity by counting pairs of samples that are consistently co-clustered or separated, with an adjustment for random agreement. Its closed-form expression in terms of C is reported in Equation (5).

(5) ARI=∑k,ℓCkℓ2−∑kak2∑ℓbℓ2/N212∑kak2+∑ℓbℓ2−∑kak2∑ℓbℓ2/N2

ARI ranges in [−0.5,1], where 0 indicates random clustering, 1 denotes perfect agreement, and negative values indicate worse-than-random partitions.

Instead, AMI quantifies the information shared between partitions, normalized and corrected for chance. The definition used in our evaluation is given in Equation (6).

(6) $$\mathrm{AMI}=\frac{\mathrm{MI}(Z,Y)-E[\mathrm{MI}]}{\max\{H(Z),H(Y)\}-E[\mathrm{MI}]}$$

MI(Z,Y) denotes the mutual information between the two partitions and H(Z), H(Y) are their entropies. E[MI] denotes the expected mutual information under a random labeling model. In terms of the contingency matrix, mutual information is computed as in Equation (7).

(7) $$\mathrm{MI}\left(Z,Y\right)=\sum_{k=1}^{K}\sum_{\ell =1}^{K}\frac{C_{k\ell }}{N}\log\frac{N\cdot C_{k\ell }}{a_{k}\cdot b_{\ell }}$$

AMI typically ranges in [0,1], with higher values indicating better clustering quality.

Both ARI and AMI are preferred over their unadjusted counterparts because the chance correction accounts for the similarity expected under random assignments. This makes comparisons more reliable across different clustering configurations and across datasets with varying numbers of clusters.

### 3.3. Results

First, we tested the capability of ResNet18 to extract meaningful features from the various bands. Table 1 shows the best results achieved considering each band alone or combining the bands. All the values reported here and in the following tables are the means across the fresco sets. We observe that the scores are generally low, with UVF as the strongest single band (0.42 ARI and 0.40 AMI). Spectral Clustering is the best choice in almost all the combinations, with the only exception being IR alone. Importantly, combining the bands did not provide any advantage, and even the best combination (VIS+UVF) yielded lower scores than using UVF alone.

We then tested the handcrafted features. We started by considering each band alone. Table 2 and Table 3 show the results obtained for the binary and ternary cases, respectively. For each band, we reported the best scores achieved with each separate feature and the best scores achieved by combining the features. In both cases, we noticed a clear improvement in the results with respect to using the features extracted via deep learning (Table 1). Again, UVF appeared to be the most informative band (0.77 ARI and 0.78 AMI), outperforming all the others. As for the features, those based on histogram and Gabor appeared to be the most significant, while LBP performed poorly in all the bands. As for the clustering, Spectral Clustering was the best clustering method overall.

Finally, we tested all the possible combinations of bands and handcrafted features. Table 4 reports the three best-performing combinations for both binary and ternary clustering. For all of them, the best-scoring clustering algorithm is Spectral Clustering. The results show a clear improvement with respect to using each band alone. For the binary case, the ARI grows from 0.77 up to 0.84 and the AMI from 0.78 up to 0.83. The improvement is even more pronounced in the ternary case: the ARI grows from 0.59 up to 0.73 and the AMI from 0.64 up to 0.74. Again, UVF provides the largest contribution in both settings.

**Figure 6:**
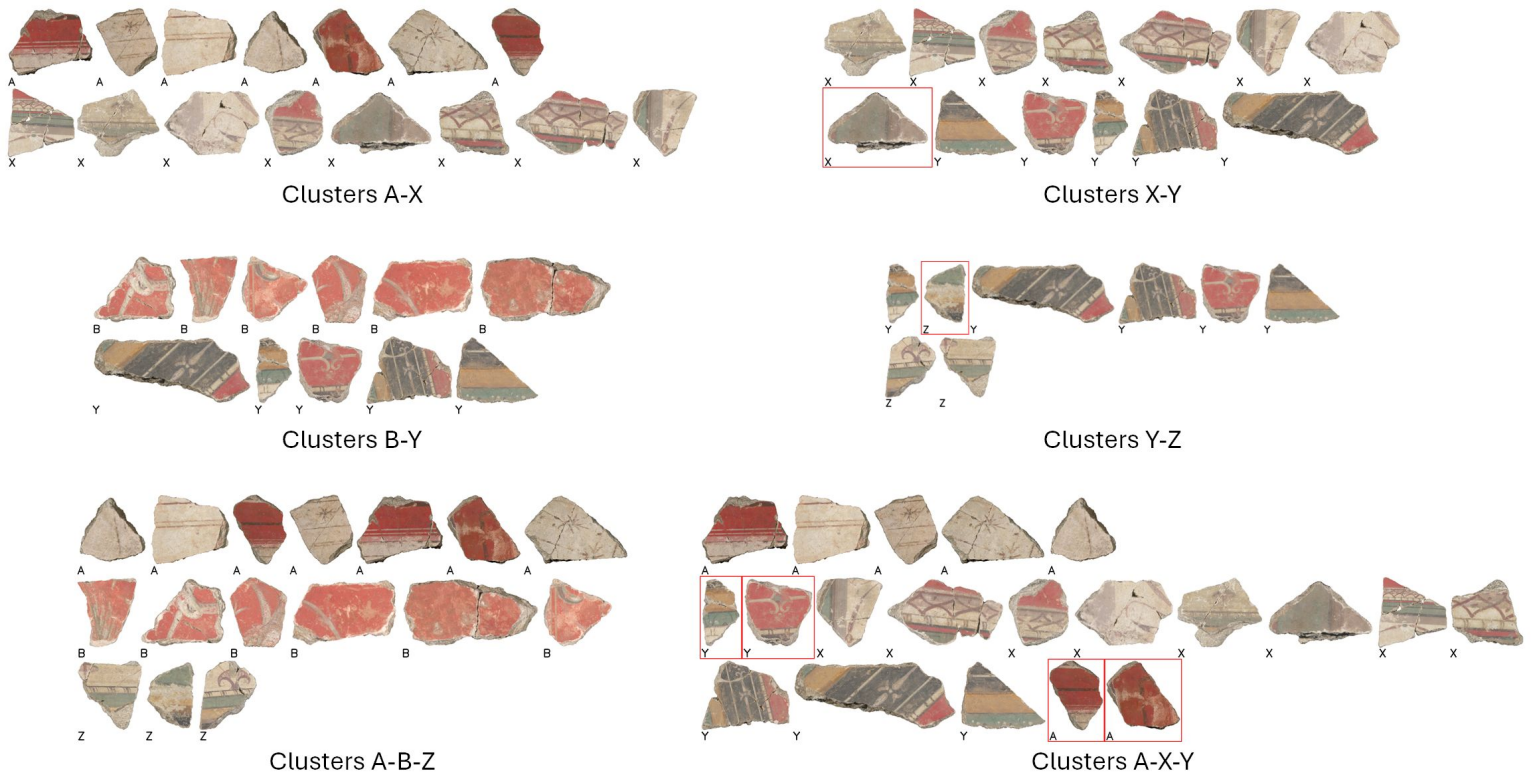
Examples of correct (**left** column) and incorrect (**right** column) clustering using the best configurations of bands and features. The letters under the fragments are the correct labels; wrongly placed fragments are highlighted with a red box. Only VIS images are reported for simplicity.

Figure 6 shows some examples of correct and incorrect clustering using the best overall configurations. The left column shows examples in which the multi-band data helped to perfectly separate the clusters, while the right column shows examples in which some errors persist. We can see how combining the bands helps to correctly discriminate ambiguous situations that appear very similar in the visible band. For example, in the pair A-X, a whitish fragment is properly assigned to cluster X, despite the presence of various whitish fragments in cluster A. A similar situation occurs for the pair B-Y where a reddish fragment is correctly assigned to cluster Y, despite the presence of various reddish fragments in B. As for the errors, in the binary case, we have at most one misplacement, while, in the ternary case, the errors grow, especially in the most challenging cases, like the triplet A-X-Y, in which some features happen to be similar between fragments of different clusters.

#### Multi-Band Versus Single-Band Configurations

The previous experiments highlighted the best overall combinations. We now want to verify if the multi-band configurations provide advantages over the single-band ones, independently of the clustering algorithm used.

**Figure 7:**
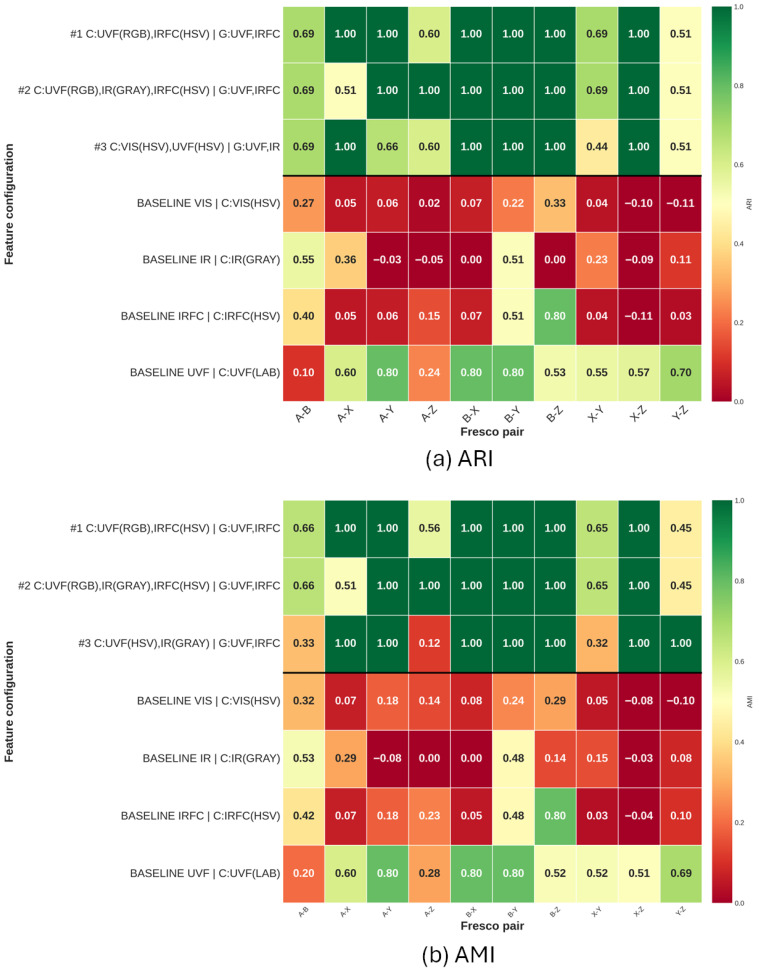
Performance matrices for the binary clustering case. The upper block reports the Top Three multi-band feature configurations (ranked by the corresponding mean score), while the lower block shows the best single-band baselines (separated by the horizontal line). Each cell contains the mean performance value for a given fresco pair. Configuration labels follow the notation C: band-wise color histogram descriptors with the adopted color channel in parentheses, and G: bands on which Gabor texture features are extracted.

**Figure 8:**
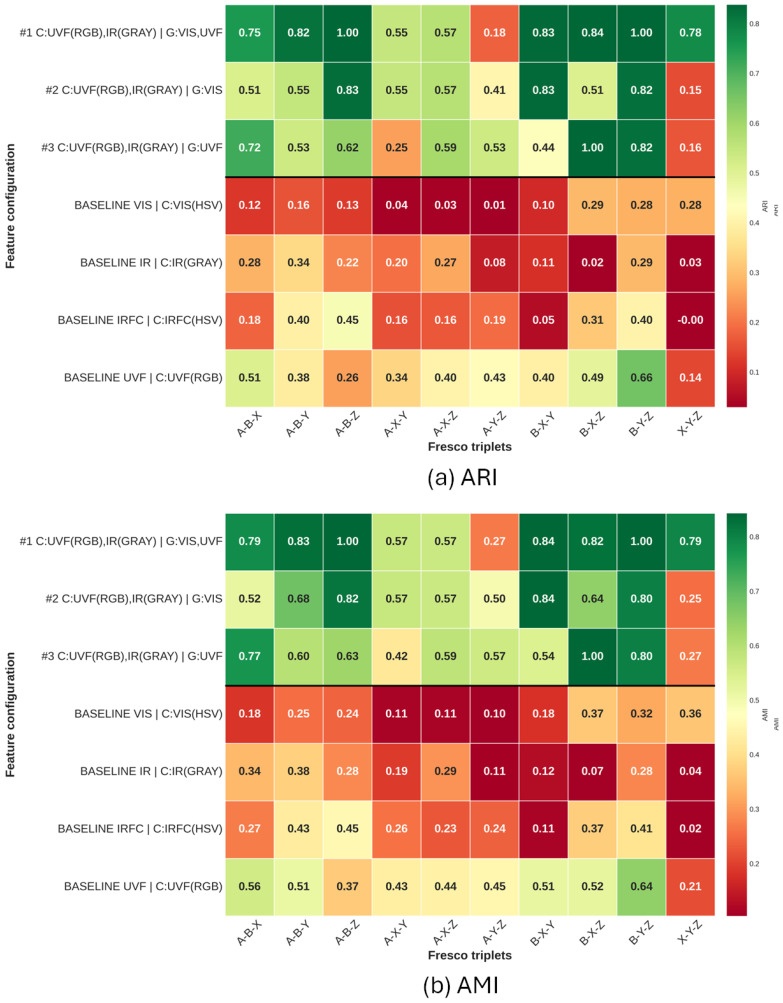
Performance matrices for the ternary clustering case. The upper block reports the Top Three multi-band feature configurations (ranked by the corresponding mean score), while the lower block shows the best single-band baselines (separated by the horizontal line). Each cell contains the mean performance value for a given fresco pair. Configuration labels follow the notation C: band-wise color histogram descriptors with the adopted color channel in parentheses and G: bands on which Gabor texture features are extracted.

To facilitate discussion of the large combinatorial experimental space, we summarized ARI and AMI scores in the performance matrices shown in Figure 7 (binary) and Figure 8 (ternary). Rows correspond to feature configurations (combinations of color histogram, Gabor, and LBP descriptors computed on the VIS, IR, IRFC and UVF bands) and columns correspond to the evaluated *fresco sets*, including both pairs and triplets. Each cell reports a clustering quality score (ARI or AMI). For a configuration *c* and fresco set *p*, results are aggregated as the mean metric value over the available clustering methods, yielding K(c,p). The matrix, therefore, provides a compact overview of cross-set robustness, i.e., whether a configuration improves clustering consistently across diverse fresco sets or only in a subset of cases. The displayed configurations are selected to represent two distinct methodological regimes. The upper section contains the three best-performing multi-band combinations, ranked by the global mean score across all fresco sets, as reported in Equation (8).

(8) $$\bar{K}\left(c\right)=E_{p}\;\left[K(c,p)\right].$$

Rankings are computed separately because ARI and AMI capture complementary aspects of clustering quality. Below the top-performing multi-band configurations, we report single-band baselines (VIS, IR, IRFC, UVF), meaning the configuration that maximizes the corresponding global mean score, thereby enabling a direct comparison between the multi-band solutions and the best achievable performance using single bands.

Across both metrics, the binary-case matrices (Figure 7a,b) show a pronounced advantage for multi-band feature combinations: the best configurations yield near-saturated scores on most fresco pairs, while single-band VIS and IR baselines remain consistently low and occasionally negative. Among the baselines, UVF is clearly the strongest single band; yet it remains more variable across pairs than the best multi-band configurations. Importantly, IRFC appears complementary rather than dominant on its own: although the IRFC baseline is generally weaker than UVF, it provides strong gains for specific pairs (e.g., the high IRFC cell in the pairwise matrices) and, when combined with UVF and texture descriptors, contributes to more uniformly high performance.

The ternary-case matrices (Figure 8a,b) highlight the expected increase in difficulty when moving from binary to ternary mixtures. Even the top multi-band configurations remain strong on several triplets, but the score distribution becomes noticeably broader, with some triplets exhibiting markedly lower ARI/AMI than in the pairwise case. The same qualitative hierarchy persists: UVF remains the best single-band baseline overall, VIS and IR are consistently weaker, and IRFC provides selective improvements that are most effective when used in multi-band combinations.

We can also see that, in both binary and ternary cases, the results involving set Z are the most unstable overall. This is due to the fact that set Z is the smallest one, including only three fragments; thus, even a single wrong fragment placement may significantly reduce the scores (see, for example, case Y-Z in Figure 6, right).

Taken together, Figure 7 and Figure 8 support two main conclusions: (i) UVF carries the most discriminative information for clustering these fragments and (ii) combining UVF with complementary bands (notably IR/IRFC) and texture cues yields the most robust behavior, especially when the task becomes more challenging in the ternary setting.

## 4. Discussion

The experiments showed that MSI data can significantly improve the clustering results with respect to using VIS images alone. While Spectral Clustering is the best performing method overall (Table 2, Table 3 and Table 4), the advantage of combining bands is independent of the clustering method employed (Figure 7 and Figure 8).

Specifically, UVF images prove to be the most informative. Even when used alone, they can reach scores close to 0.8 for both ARI and AMI, a result far higher than those obtained by all the other bands, either when used alone or in combination. Moreover, all the best combinations include both histogram and Gabor UVF features, further stressing the importance of this component. IRFC is the second-best image type. When used alone it can only slightly improve the results with respect to VIS data alone, but it can make a significant contribution to refining the results when used in combination with UVF. As expected, IR alone cannot provide enough information for good clustering, but, like IRFC, it can help improve the scores when combined with VIS and UVF.

Even if we did not directly employ the materials’ spectral responses for the clustering, the obtained outcomes indirectly depend on the different compositions of the materials, as well as surface alterations or degradations. For example, the predominant influence of UVF images may depend on differences in the surface composition that produce more discriminative fluorescence colors or shades under UV light. On the other hand, it is also possible that superficial details may appear more evident in UVF images than in VIS images due to their absence of fluorescence (e.g., as dark spots near a colored area). Similar consideration can be applied to IR/IRFC images as well.

Regarding the feature extraction methodologies, the deep learning approach seems not to be promising, giving low performances in all the combinations. This is likely due to the small dimensions of the fragments and the absence of complex pictorial content; thus not much information can be extracted by the CNN. Handcrafted features prove to be more effective in this scenario; specifically, histogram-based and Gabor features are always present in all the best combinations. On the contrary, LBP features underperform, providing lower scores overall. This behavior can again be attributed to the characteristics of the fragments: they present vivid colors and geometrical patterns that are well captured by histograms and Gabor respectively but do not exhibit recurrent local patterns that can be exploited by LBP. Overall, these results seem to highlight that, when dealing with archeological wall painting, a small number of carefully selected features can provide better results than more complex approaches.

However, even with the best combination of features and bands, we can still observe a significant drop in performance when moving from binary to ternary clustering. This behavior shows the intrinsic complexity of the problem, even with a small number of elements to group. In fact, archeological wall painting fragments are generally difficult to separate. They often show only a few colors and simple geometrical patterns that can present features very similar to each other, while it is rarer to find wall paintings with well preserved and complex pictorial contents (like in the exceptional case of Pompeii).

It must be stressed that this study is intended only as a preliminary exploration of the MSI technique for the clustering of archeological wall painting fragments. Given the small size of the available dataset, we cannot reach a general solution and provide the best overall combination of parameters that are equally valid for both binary and ternary cases. It is possible that, given more data, a better combination of features able to reduce the gap between the binary and ternary clustering may arise. Despite this limitation, the results still give us some interesting clues about the properties and the effectiveness of the MSI data for this task. The combined use of multiple bands provides a huge improvement in performance, doubling both ARI and AMI scores with respect to the results achievable using VIS image alone. While not conclusive, we think that such behavior is very promising, showing some interesting directions for future studies.

## 5. Conclusions

In this paper, we present an exploratory study on the use of multispectral imaging for the automatic clustering of archeological wall painting fragments. Experiments conducted on a set of ancient Roman fragments indicate that leveraging multiple multispectral modalities can improve clustering performance in both binary and ternary settings compared with using visible imaging alone. Among the considered modalities, UV-induced fluorescence provides the most discriminative information overall, followed by infrared false color.

Future work will focus on acquiring a larger dataset of fragments to validate these findings and assess their generality. A larger dataset will also allow us to test more complex deep learning strategies—such as domain-specific fine-tuning, self-supervised learning, or patch-based training—that require a large amount of data to properly work. We also plan to investigate additional spectral bands as well as alternative handcrafted feature representations and clustering strategies.

More broadly, the proposed framework could be transferred to other classes of cultural heritage artifacts, such as ceramics or mosaics, although its effective application would likely require adaptation to the specific visual, texture, and material properties of the analyzed objects.

## Figures and Tables

**Table 1:** Results (as means across all wall painting sets) using ResNet18 as feature extractor: best scores for each separate band and for best combination of bands. Best overall results in bold; second-best underlined.

Case	Band	Clustering	ARI	AMI
Binary	VIS	Spec. Clust.	0.2190	0.2592
	IR	Spec. Clust.	0.0597	0.0573
	IRFC	Spec. Clust.	0.1765	0.1715
	UVF	Spec. Clust.	**0.4212**	**0.4018**
	VIS + UVF	Spec. Clust.	0.4180	0.4286
Ternary	VIS	Spec. Clust.	0.2123	0.2480
	IR	BIRCH	0.1188	0.1309
	IRFC	Spec. Clust.	0.1562	0.1565
	UVF	Spec. Clust.	**0.3698**	**0.4130**
	VIS +UVF	Spec. Clust.	0.3339	0.3725

**Table 2:** Results (as means across all wall painting sets) for the binary case using the handcrafted features: best scores for each separate band considering each feature alone and the best combination of features. Best overall results per-band in bold; second-best underlined.

Band	Features	Clustering	ARI	AMI
VIS	${\mathrm{Hist}}_{HSV}$	Spec. Clust.	**0.3612**	**0.3821**
	LBP	Spec. Clust.	0.0437	0.0302
	Gabor	Spec. Clust.	0.0621	0.0956
	${\mathrm{Hist}}_{HSV}$ + Gabor	Spec. Clust.	0.2541	0.3294
IR	${\mathrm{Hist}}_{Gray}$	Spec. Clust.	0.1854	0.2172
	LBP	Spec. Clust.	0.0228	0.0035
	Gabor	Spec. Clust.	**0.2622**	**0.2652**
	${\mathrm{Hist}}_{Gray}$ + Gabor	K-means	0.2112	0.2147
IRFC	${\mathrm{Hist}}_{HSV}$	Spec. Clust.	0.3263	0.3657
	LBP	Spec. Clust.	0.0390	0.0265
	Gabor	Spec. Clust.	0.2567	0.2763
	${\mathrm{Hist}}_{RGB}$ + Gabor	Ward	**0.4061**	**0.4331**
UVF	${\mathrm{Hist}}_{HSV}$	Spec. Clust.	0.6849	0.6589
	LBP	Spec. Clust.	0.0390	0.0265
	Gabor	BIRCH	0.5821	0.6073
	${\mathrm{Hist}}_{LAB}$ + Gabor	Spec. Clust.	**0.7727**	**0.7852**

**Table 3:** Results (as means across all wall painting sets) for the ternary case using the handcrafted features: best scores for each separate band considering each feature alone and the best combination of features. Best overall results per-band in bold; second-best underlined.

Band	Features	Clustering	ARI	AMI
VIS	${\mathrm{Hist}}_{LAB}$	Spec. Clust.	0.3185	0.3952
	LBP	BIRCH	0.0210	−0.0152
	Gabor	Spec. Clust.	0.0515	0.0823
	${\mathrm{Hist}}_{LAB}$ + Gabor	Spec. Clust.	**0.3942**	**0.4429**
IR	${\mathrm{Hist}}_{Gray}$	Spec. Clust.	0.2159	0.2864
	LBP	K-means	0.0182	−0.0149
	Gabor	Spec. Clust.	0.1699	0.1804
	${\mathrm{Hist}}_{Gray}$ + Gabor	BIRCH	**0.2683**	**0.2945**
IRFC	${\mathrm{Hist}}_{HSV}$	Spec. Clust.	**0.3254**	**0.3960**
	LBP	BIRCH	0.0272	−0.0044
	Gabor	Spec. Clust.	0.1176	0.1434
	${\mathrm{Hist}}_{HSV}$ + Gabor	Spec. Clust.	0.2984	0.3346
UVF	${\mathrm{Hist}}_{RGB}$	Spec. Clust.	0.4899	0.5669
	LBP	Ward	0.0263	−0.0031
	Gabor	Spec. Clust.	0.5724	0.6430
	${\mathrm{Hist}}_{LAB}$ + Gabor	Spec. Clust.	**0.5967**	**0.6498**

**Table 4:** Results (as means across all wall painting sets) for the Top Three combinations of bands and features for both binary and ternary cases. Spectral Clustering was used as the clustering algorithm for all.

Case	Band	Features	ARI	AMI
Binary	UVF + IRFC	${\mathrm{Hist}}_{RGB}$(UVF) + Gabor(UVF) +	0.8489	0.8313
		${\mathrm{Hist}}_{HSV}$(IRFC) + Gabor(IRFC)		
	UVF + IR + IRFC	${\mathrm{Hist}}_{RGB}$(UVF) + Gabor(UVF) +	0.8394	0.8267
		${\mathrm{Hist}}_{HSV}$(IRFC) + Gabor(IRFC) +		
		${\mathrm{Hist}}_{Gray}$(IR)		
	VIS + UVF + IR	${\mathrm{Hist}}_{HSV}$(UVF) + Gabor(UVF) +	0.7898	0.7612
		${\mathrm{Hist}}_{HSV}$(VIS) + Gabor(IR) +		
Ternary	VIS + IR + UVF	${\mathrm{Hist}}_{RGB}$(UVF) + Gabor(UVF) +	0.7310	0.7489
		${\mathrm{Hist}}_{Gray}$(IR) + Gabor(VIS)		
	UVF + IRFC	${\mathrm{Hist}}_{RGB}$(UVF) + Gabor(UVF) +	0.7061	0.7159
		Gabor(IRFC)		
	IR + IRFC + UVF	${\mathrm{Hist}}_{RGB}$(UVF) + Gabor(UVF) +	0.6788	0.6792
		Gabor(IR) + Gabor(IRFC)		

## Data Availability

The dataset will be released in an open access format in the near future (at https://vision.unipv.it/dataset.html, accessed on 25 March 2026) upon receiving the final authorization from the local heritage authorities.

## Appendix A. Feature Extractor Specifications

This appendix provides a formal description of all of the feature extraction methods used, as listed in Section 2. The chosen hyperparameters are reported accordingly.

### Appendix A.1. Deep Learning-Based Feature Extraction

This approach works by extracting high-level semantic representations using a pretrained Convolutional Neural Network (CNN) as a feature extractor. Specifically, we employed a ResNet-18 [46] pretrained on ImageNet [47], leveraging the transferability of deep representations learned from natural images to fresco fragment analysis. The CNN was modified by removing the final fully connected layer, yielding a mapping $F:R^{224\times 224\times 3}\to R^{512}$.

During feature extraction, each fragment image I(i,s) underwent standard ImageNet preprocessing, including resizing to 224×224 and channel-wise normalization with μ=[0.485,0.456,0.406] and σ=[0.229,0.224,0.225]. To reduce background influence while preserving the fixed input shape required by the network, background pixels were replaced with the ImageNet RGB mean prior to inference. In this way, background regions were prevented from contributing to the extracted features, without introducing artificial discontinuities.

The per-band feature $\phi_{\mathrm{ResNet}}$ was obtained through the feature extractor F as in Equation (A1).

(A1) $$\phi_{\mathrm{ResNet}}\;\left(I(i,s)\right)=F\;\left(I(i,s)\right)\in R^{512}$$

### Appendix A.2. Color Histogram Descriptors

For each fragment *i* and band s∈S, color information is captured via marginal-intensity histograms computed independently on each channel of a selected color space c∈{RGB,HSV,LAB,GRAY}, using B=16 bins per channel.

Let $C_{c}$ denote the number of channels of color space *c* ($C_{c}=3$ for c∈{RGB,HSV,LAB} and $C_{c}=1$ for c=GRAY), and let $I^{\left(k\right)}\left(i,s\right)$ denote the *k*-th channel of I(i,s) with $k\in \{1,\ldots ,C_{c}\}$. We denote by $h_{i,s}^{\left(k\right)}\in R^{B}$ the resulting *B*-bin intensity histogram of channel *k* for fragment *i* in band *s*. The per-band color feature is then obtained by concatenation, according to Equation (A2).

(A2) $$\phi_{\mathrm{hist}}\;\left(I(i,s)\right)=\overset{C_{c}}{\underset{k=1}{∥}}h_{i,s}^{\left(k\right)}\;\in \;R^{C_{c}B}$$

### Appendix A.3. Local Binary Pattern (LBP) Descriptors

Local texture features are characterized using Local Binary Patterns [48]. Each fragment image is converted to grayscale and processed with the LBP operator, which encodes local structure by comparing the center pixel intensity with that of P=8 neighbors sampled on a circle of radius R=1. We adopt the uniform rotation-invariant variant, which groups rotation-equivalent uniform patterns and assigns all non-uniform patterns to a single bin. The aggregation step is summarized in Equation (A3), where LBP codes, computed on band *s* for fragment *i*, are pooled into a histogram over the fragment region.

(A3) $$\phi_{\mathrm{LBP}}\;\left(I(i,s)\right)\;=\;\mathrm{hist}\;\left({\mathrm{LBP}}_{P,R}\;\left(I(i,s)\right)\right)\;\in \;R^{P+2}$$

hist(·) denotes the L1-normalized histogram of LBP labels computed over the pixels belonging to fragment *i* (i.e., over its support). The resulting P+2 bins correspond to P+1 uniform patterns plus one non-uniform class, yielding a compact rotation-invariant texture representation.

### Appendix A.4. Gabor Filter Bank Descriptors

Gabor filters are employed to capture multi-orientation texture and edge information. Each fragment image is converted to grayscale and processed using a Gabor filter bank. Specifically, we adopt a kernel size of 31×31, $N_{\theta }=8$ orientations uniformly sampled in [0,π), aspect ratio γ=1.0, phase offset ψ=0, and multiple values of the scale parameter σ and wavelength λ.

Let ${\left\{G_{k}\right\}}_{k=1}^{M}$ denote the adopted Gabor filter bank. Equation (A4) summarizes the per-filter summary statistic: each filter response is computed by convolution and reduced to its mean squared energy over the fragment region.

(A4) $$\phi_{\mathrm{Gabor}}\;\left(I(i,s)\right)\left[k\right]=E\;\left[{\left((G_{k}*I\left(i,s\right))\left(p\right)\right)}^{2}\right]\in R^{M},\;k=1,\ldots ,M$$

The final Gabor descriptor is then formed by stacking the *M* energies into a single vector.
